# Circulating thyrotropin receptor messenger ribonucleic acid is not an effective marker in the follow-up of differentiated thyroid carcinoma

**DOI:** 10.1186/s13044-015-0024-4

**Published:** 2015-08-04

**Authors:** Surasawadee Ausavarat, Jiraporn Sriprapaporn, Busara Satayaban, Wanna Thongnoppakhun, Aunchalee Laipiriyakun, Boontham Amornkitticharoen, Rujaporn Chanachai, Chaveevan Pattanachak

**Affiliations:** Nuclear Chemistry Laboratory, Division of Nuclear Medicine, Department of Radiology, Faculty of Medicine Siriraj Hospital, Mahidol University, Bangkok, 10700 Thailand; Division of Molecular Genetics, Department of Research and Development, Faculty of Medicine Siriraj Hospital, Mahidol University, Bangkok, 10700 Thailand

**Keywords:** TSHR mRNA, Thyroid mRNA, qPCR, DTC

## Abstract

**Background:**

Circulating thyrotropin receptor messenger ribonucleic acid (TSHR mRNA) assay has been validated in the follow-up of differentiated thyroid carcinoma (DTC) because of its high sensitivity during thyroid hormone therapy and no interference with endogenous anti-thyroglobulin antibodies (TgAb) compared to serum thyroglobulin (Tg). We investigated the efficacy of TSHR mRNA assay in 160 DTC patients using quantitative PCR (qPCR).

**Findings:**

Only TSHR mRNA level of structural persistent disease with TgAb-positive (3.47 (2.97–9.53) pg equivalents/μg total RNA; *p* = 0.013) and its subgroup of distant metastasis patients with TgAb-positive (5.55 (3.28–12.52) pg equivalents/μg total RNA; *p* = 0.009) were significantly different from patients with no evidence of disease (2.32 (1.44–3.94) pg equivalents/μg total RNA). Applying cutoff at 2.00 pg equivalents/μg total RNA enabled us to predict structural persistent disease patients with a sensitivity of 62.3 % and a specificity of 42.9 %. Although, the sensitivity of TSHR mRNA assay in TgAb-postive patients (88.2 %) was superior than serum Tg (47.1 %) (*p* = 0.00002), the accuracy of the test is only 54.5 %.

**Conclusions:**

This study demonstrated that TSHR mRNA assay has good sensitivity in TgAb-positive patients but it is neither specific enough as a first-line of testing nor a surrogate marker in the follow-up of our DTC patients.

## Findings

### Introduction

Serum thyroglobulin (Tg) is widely accepted as a specific tumor marker for detection of residual, recurrence, or metastatic disease in the follow-up of patients with DTC after total thyroidectomy and ablative radioiodine therapy [1]. Due to limited usefulness of serum Tg by the possible interference of endogenous TgAb and low sensitivity during thyroid hormone suppression [2], several investigators have developed assays to detect Tg mRNA from circulating thyroid cells in patients with DTC. The study on efficacy of Tg mRNA in circulation as a novel tumor marker for DTC was first reported in 1966 by Ditkoff BA, et al. [3]. Tg mRNA were the most studied, however, the results have been variable from “significant” to “no such correlation” of these thyroid specific mRNAs and the presence of disease [4]. The sensitivity of Tg mRNA ranged from 25 % [5] to 100 % [6] and the specificity ranged from 24.2 % [7] to 95.8 % [8]. The discrepancies of these results were due to different methods of detection and quantification of Tg mRNAs. Later on, many studies depended on the use of qPCR based techniques which basically gave higher sensitivity than reverse transcription PCR; therefore thyroid mRNA detection was changed from qualitative to quantitative measurement [9]. A cutoff limit has been applied for the presence of thyroid carcinoma since this mRNA was detected in normal subjects and in patients with benign thyroid diseases [9]. Other thyroid specific mRNAs have been studied, for instance, thyrotropin receptor (TSHR) mRNA [10], sodium iodide symporter (NIS) mRNA [11] and thyroid peroxidase (TPO) mRNA [12]. The most comprehensive study is from Cleveland Clinic which determined the TSHR mRNA in monitoring of DTC and extended to benign thyroid diseases [13]. The study has shown so much promise that, nowadays, it has been introduced into routine clinical practice as a surrogate marker for circulating thyroid cells. In this study, we quantify TSHR mRNA by employing qPCR and evaluate the efficacy of TSHR mRNA in the follow-up of 160 patients with DTC.

**Fig. 1:**
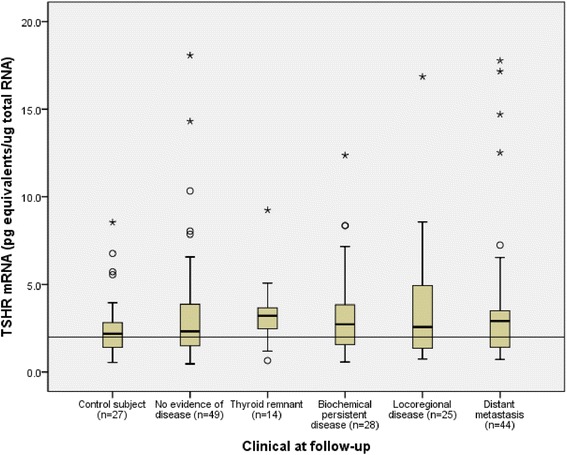
TSHR mRNA level of each study group during thyroid hormone therapy. Data is represented as interquartile range with median of each group in boxplot. Circles and asterisks are outlier and extreme data, respectively. The cutoff for assay positivity is at 2.00 pg equivalents/μg total RNA

## Patients and methods

### Patients

We evaluated 160 patients with DTC treated at our Thyroid Clinic, Division of Nuclear Medicine, Department of Radiology, Faculty of Medicine Siriraj Hospital as well as 27 normal subjects without history of thyroid diseases as controls. All DTC patients underwent total or near-total thyroidectomy and at least one series of radioactive iodine ablation (30–200 mCi). Retrospective chart review was carried out to obtain each patient’s medical history: pathological reports and all available radiological examinations. The study was approved by the Ethic Committee of the Faculty of Medicine, Siriraj Hospital. COA no. Si 442/2012. Demographic of DTC patients are shown in Table 1.

**Table 1:** Demographic of DTC patients

Characteristics		N (%)
Age (years)		
Mean ± SD	49.9 ± 14.3	
Gender		
Female		133 (83.1)
Male		27 (16.9)
Follow-up duration (years)		
Median (interquartile range)	4.0 (2.0–7.0)	
Histology		
Papillary		135 (84.4)
Follicular		17 (10.6)
Mixed		7 (4.4)
Hürthle cell		1 (0.6)
TgAb status during thyroid hormone therapy		
Positive TgAb (≥40 IU/mL)		27 (16.9)
Negative TgAb (<40 IU/mL)		133 (83.1)
Clinical status at the follow-up		
No evidence of disease (NED)		49 (30.6)
Thyroid remnants		14 (8.8)
Biochemical persistent disease		28 (17.5)
Structural persistent disease		69 (43.1)

### *In vitro* thyroid function test

All blood samples were measured for T4, TSH, Tg and TgAb on Cobas e411 platform (Roche Diagnostics GmbH, Germany) based on electrochemiluminescence immnunoassay. All DTC patients had TSH values below 2 mIU/L during thyroid hormone therapy (92.5 % had TSH below 0.5 mIU/L).

### Quantification of TSHR mRNA

Total RNA was isolated from 3 mL EDTA blood using the Geneaid kit (Geneaid, Taiwan) according to the manufacturer’s protocol. Quantitative PCR was performed using 4 μL of first-strand cDNA in a 20- l reaction volume containing 0.2 μM of each TSHR primer (Forward primer 5′-GTTCCCTGACCTGACCAAAG-3′and Reverse primer 5′-AAGGGCAGTGACACTGGTTTGAGA-3′[14]) and 10 μL of SensiFast SYBR no ROX (Bioline, MA); cycling conditions for PCR included denaturing for 2 min at 95 °C; followed by 40 cycles of 110 s at 95 °C; 15 s at 60 °C; 20 s at 72 °C; and 20 s at 72 °C; using LightCycler@480 Real-Time PCR System (Roche Diagnnostics GmbH, Germany). Each sample was assayed in duplicate. PCR-amplified TSHR cDNA fragment inserted into the plasmids were used to generate a standard curve. A calculation was made to separate the weight of the TSHR fragment from the plasmid weight. The standard curve included five different concentrations (0.00362, 0.0362, 0.362, 3.62, and 36.2 pg equivalents/μg total RNA). The result for TSHR mRNA level of each subject was calculated from the standard curve and expressed as pg equivalents/μg total RNA [6–8].

### Statistical analysis

All statistical analyses were performed with SPSS software (version 18.0; SPSS, Chicago, Illinois). Values with normal distribution were expressed as mean± (SD) and non-normal distribution were expressed as median (25th–75th percentiles). Differences of TSHR mRNA levels between investigated groups were evaluated by Mann–Whitney U-test. Receiver Operating Characteristics (ROC) curve was generated and its specificity and sensitivity were obtained. Correlation of serum Tg and TSHR mRNA was performed by Spearman’s rank correlation. A *p* value of 0.05 was considered statistically significant.

## Results

### TSHR mRNA assay

We detected TSHR mRNA in all subjects. TSHR mRNA level of each group were also classified according to their TgAb status (Fig. 1). TSHR mRNA level of structural persistent disease with TgAb-positive (3.47 (2.97–9.53) pg equivalents/μg total RNA; *p* = 0.013) and its subgroup of distant metastasis patients with TgAb-positive (5.55 (3.28–12.52) pg equivalents/μg total RNA; *p* = 0.009) were significantly different from NED (2.32 (1.44–3.94) pg equivalents/μg total RNA). Cutoff of TSHR mRNA and serum Tg were obtained from ROC curve analysis and their percent positivity are shown in Table 2.

**Table 2:** TSHR mRNA levels of the study groups

Group	TSHR mRNA median (range)^*a*^			TSHR mRNA >2.0^*a*^	Serum Tg > 0.3^*b*^
	Total	TgAb-ve	TgAb + ve		
Control subject	2.19 (1.30–2.99)	2.19 (1.30–2.99)	-	-	-
	*n* = 27	*n* = 27			
No evidence of disease	2.32 (1.44–3.94)	2.32 (1.44–3.94)	-	57.1 %	18.4 %
	*n* = 49	*n* = 49			
Thyroid remnants	3.21 (2.22-3.88)	3.05 (1.71–3.63)	4.24 (3.39–5.08)	78.6 %	7.1 %
	*n* = 14	*n* = 12	*n* = 2		
Biochemical persistent disease	2.72 (1.51–3.98)	2.42 (1.21–3.98)	3.59 ± 2.37	64.3 %	53.6 %
	*n* = 28	*n* = 20	*n* = 8		
Structural persistent disease	2.75 (1.37–3.83)	2.22 (1.13–3.28)	3.47 (2.97–9.53)^*c*^	62.3 %	79.7 %
	*n* = 69	*n* = 52	*n* = 17		
Locoregional disease	2.56 (1.19–5.03)	2.00 (1.03–4.93)	3.01 (1.89–8.16)	60.0 %	80.0 %
	*n* = 25	*n* = 19	*n* = 6		
Distant metastasis	2.92 (1.40–3.49)	2.24 (1.17–3.19)	5.55 (3.28–12.52)^*c*^	63.6 %	79.5 %
	*n* = 44	*n* = 33	*n* = 11		

^*a*^Unit of TSHR mRNA: pg equivalents/μg total RNA

^*b*^Unit of serum Tg: ng/mL

^*c*^Significance at *p* <0.05 comparing to no evidence of disease

### TSHR mRNA in TgAb-positive patients

We defined TgAb-positive when TgAb was higher than its functional sensitivity at 40.0 IU/mL. There were 27 disease persistent patients who were positive for TgAb (24.3 %) (Median TgAb = 345.9 (84.9–1207.0) IU/mL). Of these 27 patients TgAb-positive, 2 were thyroid remnants, 8 were biochemical persistent disease, and 17 were structural persistent disease. TSHR mRNA was positive for 24 out of 27 TgAb-positive patients. The levels of TSHR mRNA in TgAb-positive patients of each group tend to be higher than that of TgAb-negative patients (Table 2).

### Diagnostic performance of TSHR mRNA, Tg and ^131^I WBS

We evaluated the diagnostic performance of each test in identifying structural persistent disease (structural persistent disease, *n* = 69 *vs* NED, *n* = 49). TSHR mRNA had high sensitivity, especially in TgAb-positive patients (88.2 %) compared to the sensitivity of serum Tg in TgAb-positive patients (47.1 %). Serum Tg was highly useful in predicting distant metastasis in TgAb-negative patients with sensitivity and specificity of 90.4 % and 81.6 %, respectively. In addition, I-131 WBS had lowest sensitivity (46.4 %) but highest specificity (87.8 %) in disease detection compared to others. There is no correlation of TSHR mRNA and serum Tg or TSHR mRNA and TSH in all groups. Comparison of diagnostic performance of TSHR mRNA, serum Tg and I-131 WBS are shown in Table 3.

**Table 3:** Diagnostic performance of TSHR mRNA, serum Tg, and ^131^I uptake in identifying structural persistent disease

	Diagnostic Performance (%)								
	TSHR mRNA^*a*^			Serum Tg^*b*^			^131^I whole body scan		
	Total	TgAb-ve	TgAb + ve	Total	TgAb-ve	TgAb + ve	Total	TgAb-ve	TgAb + ve
Sensitivity	62.3	53.8	88.2	79.7	90.4	47.1	46.4	42.3	58.8
Specificity	42.9	42.9	42.9	81.6	81.6	81.6	87.8	87.8	87.8
PPV^*c*^	60.6	50.0	34.9	85.9	83.9	47.1	84.2	78.6	62.5
NPV^*d*^	44.7	46.7	91.3	74.1	88.9	81.6	53.8	58.9	86.0
Accuracy	54.2	48.5	54.5	80.5	86.1	72.7	63.6	64.4	80.3

^*a*^Using a cutoff of at least 2.00 pg equivalents/μg total RNA on thyroid hormone therapy

^*b*^Using a cutoff of at least 0.3 ng/mL on thyroid hormone therapy

^c^PPV, Positive predictive value

^d^NPV, Negative predictive value

## Conclusions

There are many advantages of thyroid mRNAs detection in the follow-up of thyroid carcinoma. Thyroid mRNAs such as Tg, TSHR, TPO mRNA has been widely used to detect recurrence or metastasis of thyroid carcinoma as this mRNA showed good sensitivity and specificity for DTC [13]. We had demonstrated that only TSHR mRNA level of structural persistent disease and its subgroup of distant metastasis patients with TgAb-positive were significantly different from NED. TSHR mRNA seems to be highly sensitive in TgAb-positive patients only. Considering TSHR mRNA assay in TgAb-positive patients, 24 out of 27 were positive for either TSHR mRNA or TgAb with sensitivity 88.2 % of and specificity of 42.9 %. Similar results were reported by Chinnapa P. et al. [10] whose study found that all three TgAb-positive patients with local disease were also positive for TSHR mRNA. Milas M. et al. [15] showed that TSHR mRNA was positive for 2 out of 3 TgAb-positive patients with sensitivity 66 % of and specificity of 88 %. In addition, our study found that two patients with distant metastasis who were positive for TSHR mRNA had undetectable serum Tg (<0.1 ng/mL) and higher than the upper limit of TgAb (>4,000 IU/mL). This demonstrated that TSHR mRNA may also be useful in the follow-up patients where both Tg and TgAb were unbeneficial. However, the overall accuracy of our TSHR mRNA assay (54.2 %) is still unsuitable for thyroid management. Low specificity of TSHR mRNA assay may be explained by the illegitimate mRNA expression in extrathyroidal tissues and peripheral blood mononuclear cells [10, 16, 17]. The poor clinical utility of TSHR mRNA assay was supported by Barzon L. et al. [18] where they reported high specificity but low sensitivity with an accuracy of 61 %. Considering the sensitivity of Tg during thyroid hormone therapy, we found that Tg cutoff at 0.3 ng/mL has sensitivity of 79.7 % in predicting structural persistent disease. Through analyzing patients with absence of TgAb, sensitivity of Tg was increased to 90.4 %. This emphasizes that serum Tg remains the most efficient marker for thyroid carcinoma management. In conclusion, TSHR mRNA assay depends on molecular technique which is much more expensive and time-consuming than serum Tg that can be tested on an automated analyzer. Moreover, superior functional sensitivity (≤0.10 ng/mL) of a second generation of Tg assay made it possible to be sensitive enough for detecting basal Tg without TSH stimulation [19]. However, if there is still TgAb interference, TgAb itself can be used as a surrogate marker. So TSHR mRNA assay may be used as an adjunctive marker in patients whose serum Tg are not reliable due to the interference of TgAb.
